# UGT1A Gene Family Members Serve as Potential Targets and Prognostic Biomarkers for Pancreatic Cancer

**DOI:** 10.1155/2021/6673125

**Published:** 2021-09-20

**Authors:** Lei Feng, Yi Wang, Jiasheng Qin, Yu Fu, Zeyi Guo, Jianmin Zhang, Guolin He, Zesheng Jiang, Xiaoping Xu, Chenjie Zhou, Yi Gao

**Affiliations:** ^1^Department of Hepatobiliary Surgery II, Zhujiang Hospital, Southern Medical University, Guangzhou 510282, China; ^2^Guangdong Provincial Research Center for Artificial Organ and Tissue Engineering, Guangzhou Clinical Research and Transformation Center for Artificial Liver, Institute of Regenerative Medicine, Zhujiang Hospital, Southern Medical University, Guangzhou 510282, China; ^3^State Key Laboratory of Organ Failure Research, Southern Medical University, Guangzhou 510282, China

## Abstract

**Background:**

Pancreatic cancer (PC) is one of the most common cancers worldwide, with high mortality. The UGT1A gene family plays important roles in pharmacology and toxicology, contributing to interindividual differences in drug disposition. However, mRNA expression and prognostic value of the UGT1A gene family in PC have not been identified.

**Methods:**

Oncomine, GEPIA2, DAVID 6.8, Metascape, Kaplan-Meier plotter, cBioPortal, GeneMANIA, TRRUST v2, TIMER, and R software were used in our study.

**Results:**

The transcriptional levels of UGT1A1/3/6/8/9/10 in PC tissues were significantly higher than those in normal tissues. These results were further validated using five pairs of PC tumor tissues and adjacent nontumor tissues. A significant correlation was found between the expression of UGT1A1/6/10 and the pathological stage of PC. PC patients with lower transcriptional levels of UGT1A1/4/5/6/10 were associated with a better prognosis. The differentially expressed UGT1A gene family functions were primarily related to the glucuronidation pathway, cytokine-cytokine receptor interactions, and the ILK signaling pathway. Our data suggest that HNF1A, AHR, and CDX2 are key transcription factors for the UGT1A gene family. Furthermore, the expression levels of UGT1A1/3/8/9/10 were positively correlated with the activities of tumor-infiltrating immune cells, especially B cells. The expression levels of UGT1A6/9 were negatively correlated with macrophage infiltration levels.

**Conclusions:**

These results suggest that the UGT1A gene family could serve as a potential prognostic biomarker and target for PC. However, future studies are required to validate our findings and promote the clinical utility of the UGT1A gene family in PC.

## 1. Introduction

Pancreatic cancer (PC) is characterized by poor prognosis, primarily associated with genetic conditions, diabetes, smoking, and obesity [1–3]. Pancreatic adenocarcinoma (PAAD) is the most common type of PC. In 2018, PC was the 13th most common cancer globally, with 458,918 new cases, and the 7th most common cause of cancer-related mortality, with 432,242 deaths [4, 5]. Early surgical resection is the most effective treatment for PC. Unfortunately, PC is most commonly discovered in the middle and late stages. Recently, numerous researchers have explored the therapeutic targets of PC, from gene and mRNA to miRNA [2, 6, 7]. However, because these are far from sufficient, it is important to explore additional therapeutic targets and prognostic biomarkers for better prognosis of PC.

UDP-glycosyltransferases (UGTs) are a superfamily of enzymes found in diverse species (including animals, fungi, bacteria, and plants). UGTs catalyze the covalent addition of sugars from nucleotide UDP-sugar donors to functional groups in a broad range of lipophilic molecules [8]. In mammals, the superfamily comprises four families: UGT1, UGT2, UGT3, and UGT8. Among them, the UGT1A gene family (including UGT1A1 and UGT1A3–UGT1A10) has important roles in pharmacology and toxicology, contributing to interindividual differences in drug disposition and cancer risk [8]. Additionally, cumulative evidence has revealed that the UGT1A gene family showed increased expression or activation in several human malignancies [9, 10] and can be induced by pathways that sense demand for detoxification and modulation of endobiotic signaling molecules [8, 11]. However, there are few related studies on the UGT1A gene family as potential therapeutic targets or prognostic biomarkers in PC.

Recent studies have reported the general expression profile and function of some UGT1A gene family members in PC [12] and other cancers [13]; however, screening suitable UGT1A gene family members as therapeutic targets or prognostic biomarkers for PC is a substantial challenge, which urgently needs to be addressed. Fortunately, with the development of second-generation gene sequencing technology and expansive database platforms, a comprehensive analysis of the UGT1A gene family members in patients with PC is possible.

In this study, we used several large public databases to perform a comprehensive analysis of the relationships between the UGT1A gene family members and the pathogenesis, tumor-infiltrating immune cells, and progression of PC. Consequently, we hope to aid clinicians in selecting appropriate therapeutic drugs and more accurately predicting long-term outcomes in patients with PC.

## 2. Materials and Methods

The main methods in this study followed the methods of previous studies [14, 15].

### 2.1. Oncomine

The Oncomine platform (http://www.oncomine.org) is a publicly accessible online cancer microarray database with a powerful set of analysis functions that compute gene expression signatures, clusters, and gene set modules, automatically extracting biological insights from the data [16, 17]. In this study, analyses of mRNA levels in the UGT1A family in PC and a normal control dataset were performed using Oncomine. The parameter settings were restricted as follows: *P* value = 0.05, fold change (FC) = 2, and data type = mRNA.

### 2.2. Clinical Samples

Five pairs of fresh PC specimens and adjacent nontumor tissues were collected from the Zhujiang Hospital, Southern Medical University (Guangzhou, China). The human specimens used for validation in this study were collected between May and June 2021. The local ethics committee approved the use of these specimens. The adjacent samples were taken at a distance of at least 5 cm from the tumor.

### 2.3. RNA Extraction and Quantitative Real-Time Polymerase Chain Reaction (qRT-PCR)

Total RNA was extracted from the tissue specimens using TRIzol (Invitrogen), and qRT-PCR was performed using SYBR Green Dye (Takara, Dalian, China) according to the manufacturer's instructions. The primer sequences of the UGT1A gene family are listed in Supplementary Table 5.

### 2.4. Gene Expression Profiling Interactive Analysis 2 (GEPIA2)

GEPIA2 (http://www.gepia2.cancer-pku.cn) is a newly developed interactive web server for analyzing RNA sequencing expression data of 9736 tumors and 8587 normal samples from the Cancer Genome Atlas (TCGA) and Genotype-Tissue Expression (GTEx) dataset projects, which provide customizable functions, such as tumor/normal differential expression analysis, profiling according to cancer type or pathological stage, patient survival analysis, similar gene detection, correlation analysis, and dimensionality reduction analysis [18]. In our study, the expression analysis of the UGT1A family in PC was performed using the expression DIY module of GEPIA2, and the parameter settings were restricted as follows: log_2_FC = 1, *P* value = 0.05, use = log_2_(TPM + 1) for log scale, jitter size = 0.04, and match = TCGA normal and GTEx data.

### 2.5. DAVID 6.8

DAVID 6.8 (https://david.ncifcrf.gov/home.jsp) is a comprehensive, functional annotation website that helps investigators better clarify the biological function of submitted genes [19]. In this study, the Gene Ontology (GO) enrichment analysis (including biological processes (BP), cellular components (CC), and molecular function (MF)) and the Kyoto Encyclopedia of Genes and Genomes (KEGG) pathway enrichment analysis of the UGT1A family were performed using DAVID 6.8.

### 2.6. Metascape

Metascape (http://metascape.org) is a free, well-maintained, user-friendly gene list analysis tool for gene annotation and analysis [20]. It is an automated meta-analysis tool used to understand common and unique pathways within a group of orthogonal target-discovery studies. In this study, Metascape was used to analyze protein-protein interaction (PPI) and molecular complex detection (MCODE) in the UGT1A family.

### 2.7. Kaplan-Meier Plotter

The Kaplan-Meier plotter (http://www.kmplot.com) is an online database containing microarray gene expression data and survival information of cancer patients derived from Gene Expression Omnibus, TCGA, and the Cancer Biomedical Informatics Grid [21]. In this study, the prognostic value of the mRNA expression of UGT1A family members was evaluated using the Kaplan-Meier plotter. The overall survival (OS) and relapse-free survival (RFS) of patients with PC were determined by dividing the patient samples into two groups based on median expression (high vs. low expression) and assessed using a Kaplan-Meier survival plot, with a hazard ratio with 95% confidence intervals and log-rank *P* value.

### 2.8. cBioPortal

The cBioPortal for cancer genomics (http://www.cbioportal.org/) is affiliated with the Memorial Sloan Kettering Cancer Center and provides information regarding the integrative analysis of complex cancer genomics and clinical profiles from 105 cancer studies in the TCGA pipeline [22]. The frequency of UGT1A family alterations (amplification, deep deletion, and missense mutations), copy number variance obtained from Genomic Identification of Significant Targets in Cancer (GISTC), and the survival between the altered and nonaltered groups was obtained according to the online instructions of cBioPortal.

### 2.9. GeneMANIA and R Software

GeneMANIA (http://www.genemania.org) is a website that provides information on physical interactions, pathways, coexpression, colocalization, and shared protein domain similarity of submitted genes [23]. In this study, we used GeneMANIA to predict the top 20 genes closely related to the UGT1A family for coexpression, physical interactions, pathways, and shared protein domain similarity of the UGT1A family and then visualized using R software.

### 2.10. TRRUST v2

TRRUST v2 (https://www.grnpedia.org/trrust/) is a manually curated database of human and mouse transcriptional regulatory networks, which includes 8444 and 6552 transcription factor- (TF-) target regulatory relationships of 800 human TFs and 828 mouse TFs, respectively [24]. In this study, we used TRRUST v2 to explore the TF targets of the UGT1A gene family in patients with PC.

### 2.11. TIMER 2.0

TIMER 2.0 (http://timer.cistrome.org/) is a comprehensive resource to systematically analyze immune infiltrates across diverse cancer types [25, 26]. In our study, we assessed the expression levels of UGT1A gene family members in PC and their correlations with tumor purity and infiltrating immune cells, including CD8+ T cells, CD4+ T cells, B cells, macrophages, and neutrophils.

## 3. Results

### 3.1. Expression of UGT1A Gene Family Members in Patients with PC

The UGT1A gene family members (except for UGT1A2P, UGT1A11P, UGT1A12P, and UGT1A13P) were retrieved from the Oncomine database. We first explored the transcriptional levels of the UGT1A gene family in PC and normal pancreatic tissues in the Oncomine database. The results shown in Supplementary 1 and Table 1 indicate that the transcriptional levels of UGT1A1, UGT1A3, UGT1A6, UGT1A8, and UGT1A9 in PC tissues were significantly elevated compared with normal pancreatic tissue. These data are consistent with Pei et al. [27] who found a significant upregulation of UGT1A1 (*P* = 4.66*E* − 7, FC = 12.962), UGT1A3 (*P* = 5.17*E* − 6, FC = 7.861), UGT1A6 (*P* = 2.76*E* − 5, FC = 12.025), UGT1A8 (*P* = 3.51*E* − 5, FC = 2.626), and UGT1A9 (*P* = 1.52*E* − 5, FC = 6.065) in PC. Logsdon et al. [28] also reported that the level of UGT1A6 (*P* = 8.41*E* − 6, FC = 6.999) in PC was significantly upregulated.

**Figure 1:**
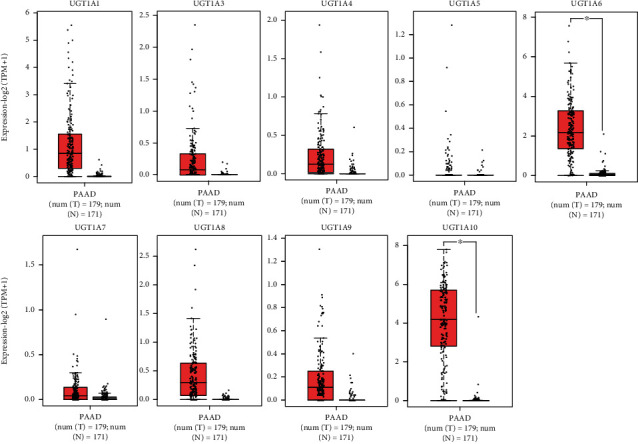
The transcription of UGT1A gene family members in patients with PC (GEPIA2). ^∗^*P* < 0.05.

Further, analysis of the expression levels of the UGT1A gene family in PC and normal tissues using GEPIA 2 showed that the transcriptional levels of UGT1A1, UGT1A3, UGT1A4, UGT1A7, UGT1A8, and UGT1A9 in PC tissues were elevated when compared with normal tissue, although there were no significant differences. Notably, the transcriptional levels of UGT1A6 (*P* < 0.05) and UGT1A10 (*P* < 0.05) in PC tissues were significantly higher than those in normal tissues (Figure 1). We also compared the relative expression levels of the U UGT1A gene family in PC tissues and found that among all UGT1A gene families evaluated, the relative expression of UGT1A10 was the highest (Supplementary 2).

**Figure 2:**
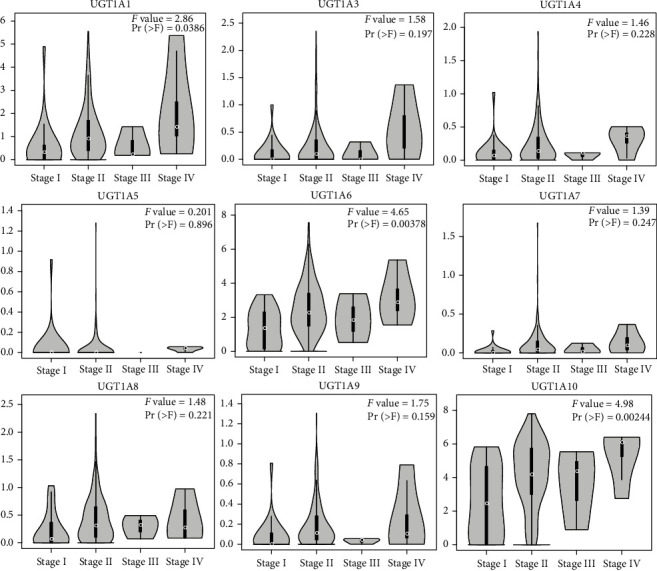
Correlation between different expressions of UGT1A gene family members and the pathological stage of patients with PC (GEPIA2).

We further assessed the correlation between the expression of the UGT1A gene family and the pathological stage of PC in patients and found a significant correlation between the expression of UGT1A1 (*P* = 0.00386), UGT1A6 (*P* = 0.00378), UGT1A10 (*P* = 0.00244), and pathological stage (Figure 2). The expression of UGT1A1, UGT1A6, and UGT1A10 increased as the tumor progressed, suggesting that the UGT1A gene family plays an important role in the tumorigenesis and progression of PC.

### 3.2. Verification of the UGT1A Gene Family in Clinical Samples

**Figure 3:**
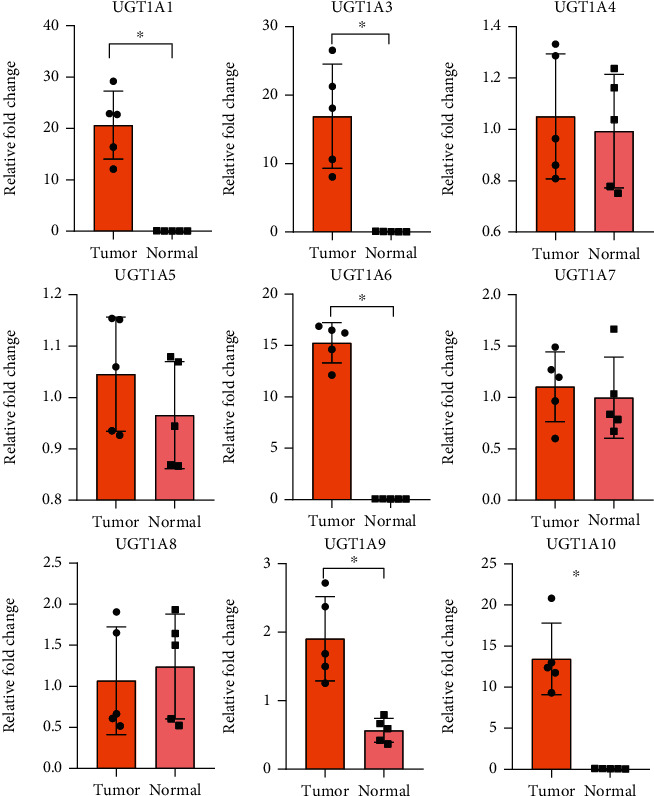
Verification of UGT1A gene family members in clinical samples. Relative mRNA levels of UGT1A gene family members in 5 PC samples were frequently overexpressed in tumor compared with matched nontumor tissues (*P* < 0.05) by qRT-PCR except UGT1A4/5/8. ^∗^*P* < 0.05.

We used qRT-PCR to assess the expression of the UGT1A gene family in five pairs of fresh tissues from PC patients and adjacent nontumor tissues. The results showed that the transcript levels of UGT1A1/3/6/9/10 were frequently higher (*P* < 0.05) in PC tissues than in the corresponding nontumor tissues (Figure 3).

### 3.3. Functional Enrichment Analysis of UGT1A Gene Family Members in Patients with PC

**Figure 4:**
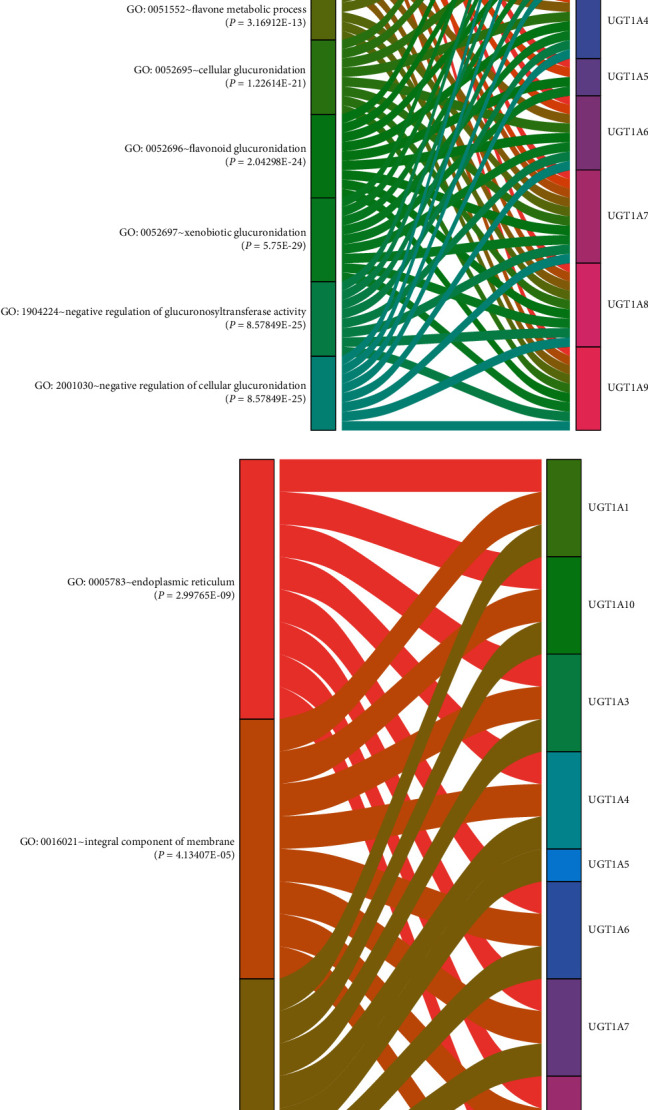
Functional enrichment analysis (BP and CC) of UGT1A gene family members in patients with pancreatic cancer (DAVID 6.8 and R software): (a) biological processes (BP); (b) cellular component (CC).

**Figure 5:**
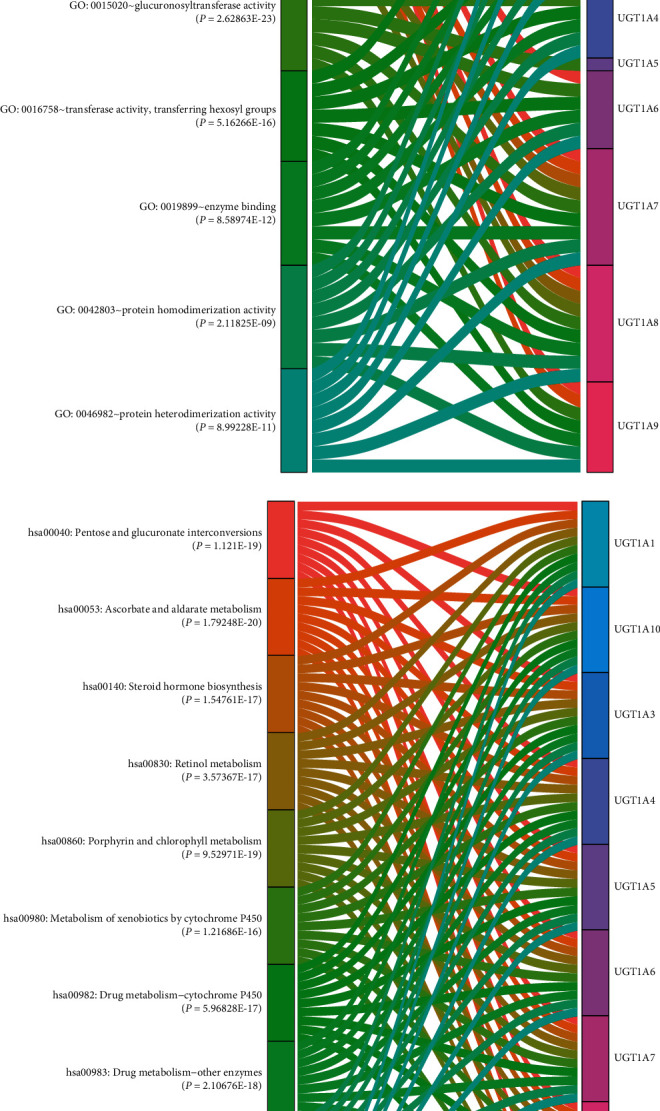
Functional enrichment analysis (MF and KEGG) of UGT1A gene family members in patients with pancreatic cancer (DAVID 6.8 and R software): (a) molecular function (MF); (b) Kyoto Encyclopedia of Genes and Genomes (KEGG).

DAVID 6.8 was utilized to analyze the functions of differentially expressed UGT1A gene family members. The UGT1A gene family members were mainly enriched in glucuronidation, metabolic regulation of BP such as xenobiotic glucuronidation, flavonoid glucuronidation, metabolic processes, negative regulation of glucuronosyltransferase activity, negative regulation of cellular glucuronidation, negative regulation of fatty acid metabolic processes, cellular glucuronidation, flavonoid biosynthetic processes, flavone metabolic processes, and retinoic acid metabolic processes. The integral components of the membrane, endoplasmic reticulum, and endoplasmic reticulum membrane were the most highly enriched items in the CC category. In the MF category, glucuronosyltransferase activity, retinoic acid binding, enzyme binding, protein heterodimerization activity, protein homodimerization activity, transferase activity, transferring hexosyl groups, enzyme inhibitor activity, steroid binding, protein kinase C binding, drug binding, and transferase activity were the 10 most highly enriched items. KEGG pathway analysis showed that among the top 10 KEGG pathways, ascorbate and aldarate metabolism, pentose and glucuronate interconversions, porphyrin and chlorophyll metabolism, drug metabolism-other enzymes, steroid hormone biosynthesis, drug metabolism-cytochrome P450, metabolism of xenobiotics by cytochrome P450, chemical carcinogenesis, and metabolic pathways were significantly associated with the tumorigenesis and progression of PC (Figures 4 and 5 and Supplementary 6).

### 3.4. PPI Network of UGT1A Gene Family Members and MCODE Component Form in the PPI Network

**Figure 6:**
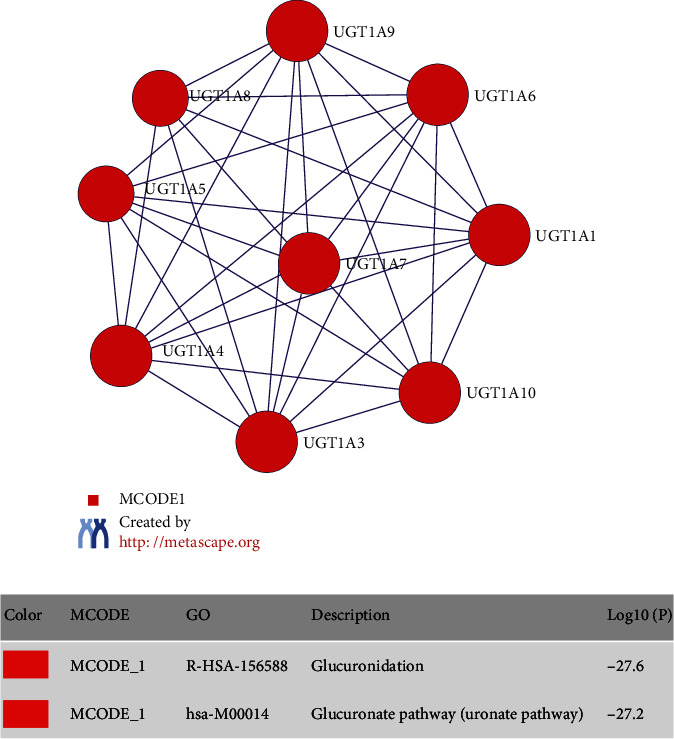
Protein-protein interaction (PPI) network of UGT1A gene family members (a) and MCODE component form in the PPI network (b).

Furthermore, to better understand the relationship between UGT1A gene family members and PC, we performed a PPI network analysis of differentially expressed UGT1A gene family members using Metascape to explore the potential interactions among them. The results showed that nine nodes and 35 edges were obtained in the PPI network (Figure 6(a)). After pathway and process enrichment analyses were independently applied to each MCODE component, the results showed that biological function was mainly related to glucuronidation and the glucuronate pathways (Figure 6(b)).

### 3.5. The Prognostic Value of UGT1A Gene Family Members in Patients with PC

**Figure 7:**
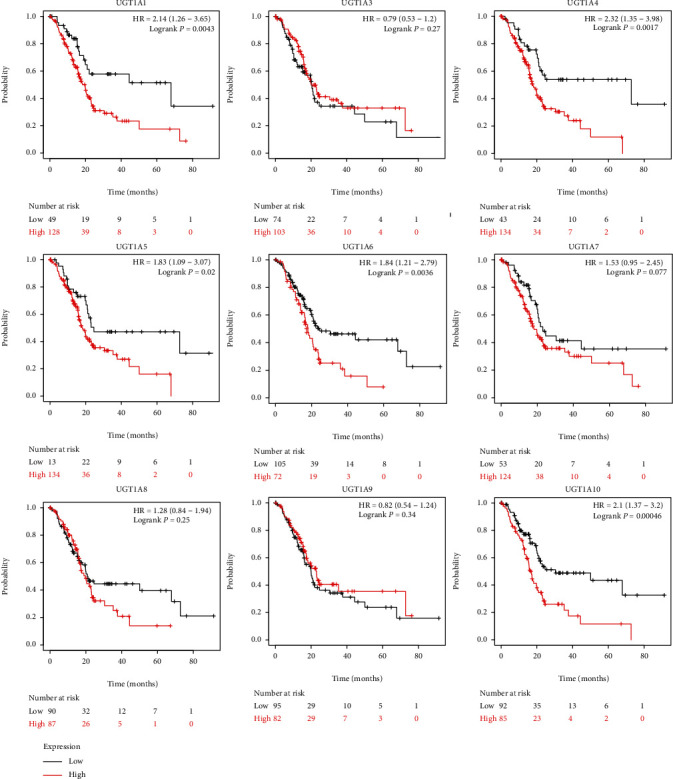
The prognostic value of differently expressed UGT1A gene family members in patients with PC in the overall survival (OS) curve (Kaplan-Meier plotter).

**Figure 8:**
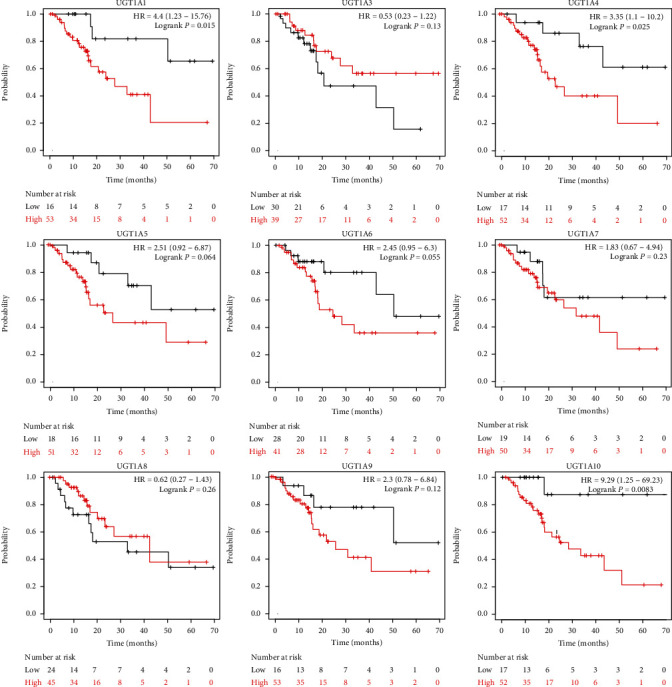
The prognostic value of differently expressed UGT1A gene family members in patients with PC in the relapse-free survival (RFS) curve (Kaplan-Meier plotter).

To evaluate the value of differentially expressed UGT1A gene family members in the progression of PC, we assessed the correlation between differentially expressed UGT1A gene family members and clinical outcomes using GEPIA 2. The value of differentially expressed UGT1A gene family members in the OS of patients with PC was evaluated. We found that PC patients with high transcriptional levels of UGT1A1 (*P* = 0.0043), UGT1A4 (*P* = 0.0017), UGT1A5 (*P* = 0.02), UGT1A6 (*P* = 0.0036), and UGT1A10 (*P* = 0.00046) were significantly associated with shorter OS (Figure 7). RFS was also assessed, and the results showed that patients with PC with high transcriptional levels of UGT1A1 (*P* = 0.015), UGT1A4 (*P* = 0.025), and UGT1A10 (*P* = 0.0083) were significantly associated with shorter RFS (Figure 8).

### 3.6. Genetic Alteration, Survival, and Interaction Analyses of UGT1A Gene Family Members in Patients with PC

**Figure 9:**
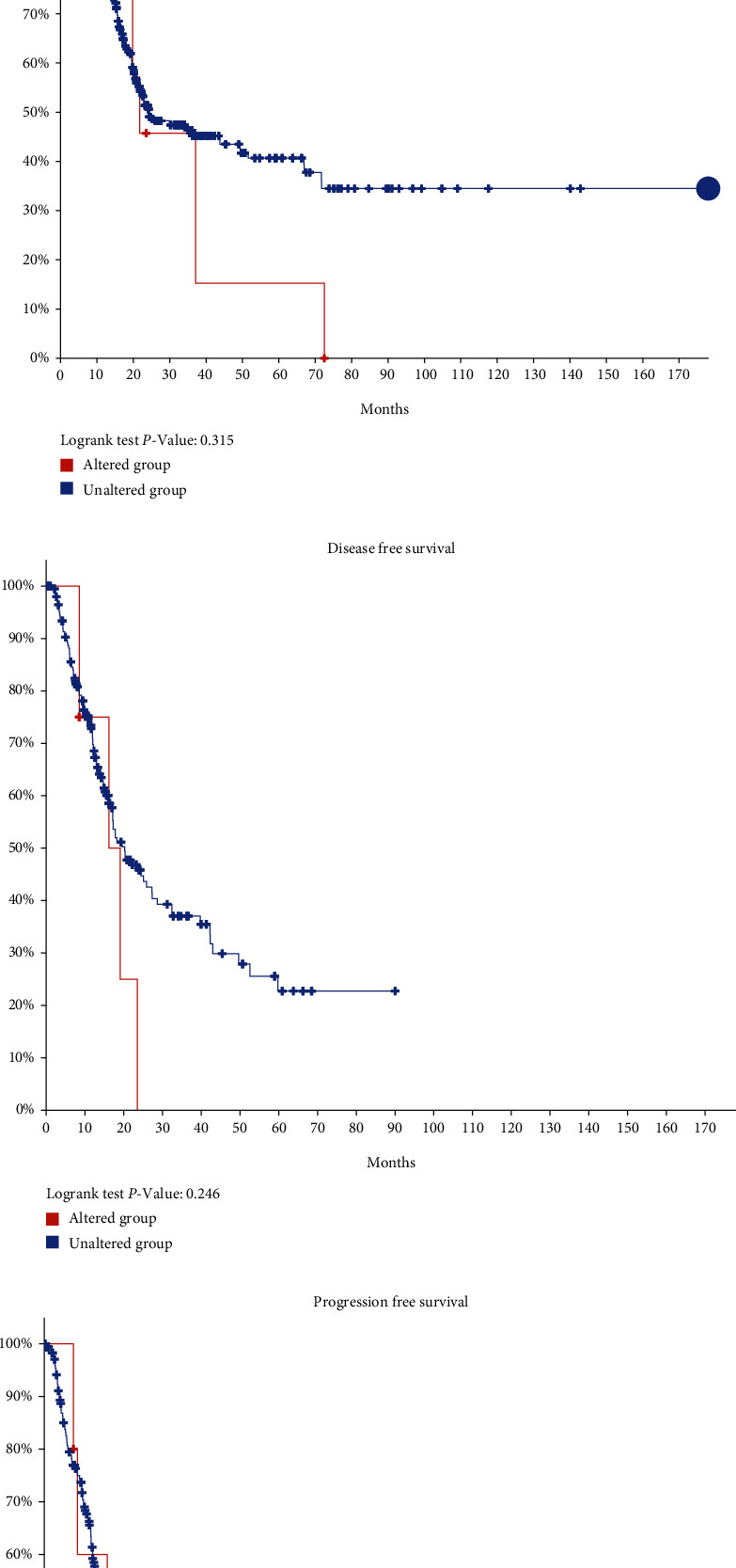
Genetic alteration (a) and survival analyses between the altered group and unaltered group (b, c, and d) of differently expressed UGT1A gene family members in patients with PC.

cBioPortal datasets from TCGA were used to analyze the genetic alterations of differentially expressed UGT1A gene family members. As a result, UGT1A1, UGT1A3, UGT1A4, UGT1A5, UGT1A6, UGT1A7, UGT1A8, UGT1A9, and UGT1A10 were altered in 0.5, 0.6, 1.1, 0.4, 0.4, 0.5, 0.6, 0.7, and 0.6% of the queried PC samples, respectively (Figure 9(a)). We further explored survival, disease-free survival (DFS), and progression-free survival (PFS) between the altered and unaltered groups. There were no significant differences in OS, DFS, and PFS between the altered and unaltered groups (Figures 9(b)–9(d)).

### 3.7. Physical Interactions, Pathways, Coexpression, and Shared Protein Domain Similarity of UGT1A Gene Family Members in Patients with PC

We used GeneMANIA to predict the top 20 genes closely related to UGT1A gene family members for coexpression, physical interactions, pathways, and protein domain similarity of the UGT1A gene family members, which were then visualized using R software. The results revealed relationships between UGT1A1 and UGT1A6, UGT1A6, and UGT1A8. Additionally, relationships were noted in physical interactions between UGT1A1 and UGT1A3, UGT1A4, UGT1A6, UGT1A7, UGT1A8, UGT1A9, and UGT1A10. In addition, shared protein domains were noted among UGT1A3 with UGT1A4, UGT1A5, and UGT1A9; UGT1A1 with UGT1A3, UGT1A4, UGT1A5, UGT1A6, UGT1A7, UGT1A8, UGT1A9, and UGT1A10 (Supplementary 3); and glucuronidation, UDP-glucuronosyl/UDP-glucosyltransferase, drug metabolism-other enzymes, retinol metabolism, steroid hormone biosynthesis, porphyrin and chlorophyll metabolism, other types of O-glycan biosynthesis, pentose and glucuronate interconversions, starch and sucrose metabolism, and ascorbate and aldarate metabolism (Supplementary 4).

### 3.8. TF Targets of UGT1A Gene Family Members in Patients with PC

Furthermore, we explored possible TF targets of the differentially expressed UGT1A gene family members using the TRRUST database. The results showed that UGT1A1, UGT1A4, UGT1A6, UGT1A7, UGT1A8, UGT1A9, and UGT1A10 were included in TRRUST. Additionally, we found that HNF1A, AHR, and CDX2 are three key TFs associated with the regulation of the UGT1A family. HNF1A is the key TF for UGT1A1, UGT1A7, UGT1A8, UGT1A9, and UGT1A10, AHR for UGT1A1 and UGT1A6, and CDX2 for UGT1A8 and UGT1A10 (Table 2).

### 3.9. Immune Cell Infiltration of UGT1A Gene Family Members in PC

**Figure 10:**
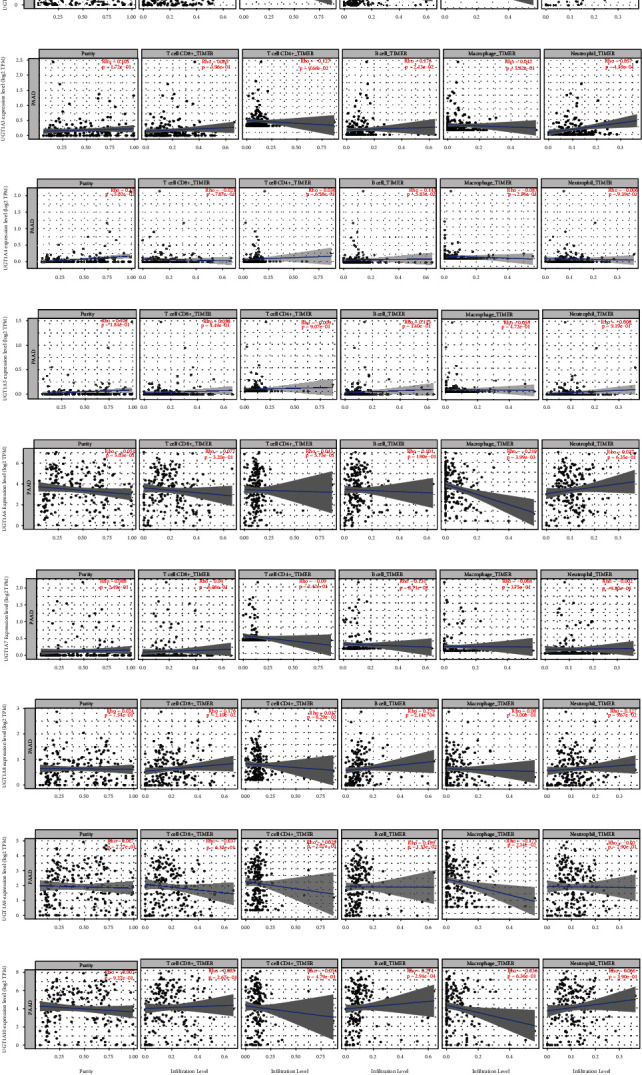
Correlations of UGT1A gene family member expression with immune infiltration level in PC (using the TIMER 2.0 database). (a) The correlation between each type of immune cells (CD8+ T cells, CD4+ T cells, B cells, macrophages, and neutrophils) and UGT1A1. (b) The correlation between each type of immune cells (CD8+ T cells, CD4+ T cells, B cells, macrophages, and neutrophils) and UGT1A3. (c) The correlation between each type of immune cells (CD8+ T cells, CD4+ T cells, B cells, macrophages, and neutrophils) and UGT1A4. (d) The correlation between each type of immune cells (CD8+ T cells, CD4+ T cells, B cells, macrophages, and neutrophils) and UGT1A5. (e) The correlation between each type of immune cells (CD8+ T cells, CD4+ T cells, B cells, macrophages, and neutrophils) and UGT1A6. (f) The correlation between each type of immune cells (CD8+ T cells, CD4+ T cells, B cells, macrophages, and neutrophils) and UGT1A7. (g) The correlation between each type of immune cells (CD8+ T cells, CD4+ T cells, B cells, macrophages, and neutrophils) and UGT1A8. (h) The correlation between each type of immune cells (CD8+ T cells, CD4+ T cells, B cells, macrophages, and neutrophils) and UGT1A9. (i) The correlation between each type of immune cells (CD8+ T cells, CD4+ T cells, B cells, macrophages, and neutrophils) and UGT1A10.

Finally, because the prognosis of PC may be affected by inflammatory response and infiltrating immune cells, we further evaluated the association between differentially expressed UGT1A gene family members and immune cell infiltration using the TIMER 2.0 database. As shown in Figure 10, a positive correlation was observed between UGT1A1 (Rho = 0.027, *P* = 6.47*E* − 3), UGT1A3 (Rho = 0.176, *P* = 2.13*E* − 2), UGT1A8 (Rho = 0.279, *P* = 2.14*E* − 4), UGT1A9 (Rho = 0.189, *P* = 1.33*E* − 2), and UGT1A10 (Rho = 0.274, *P* = 2.94*E* − 4) expression and infiltrating B cells. A positive correlation was observed between UGT1A8 (Rho = 0.176, *P* = 2.10*E* − 2) and infiltrating CD8+ T cells. Moreover, UGT1A6 (Rho = −0.219, *P* = 3.99*E* − 3) and UGT1A9 (Rho = −0.173, *P* = 2.34*E* − 2) expression was negatively correlated with infiltrating macrophages.

## 4. Discussion

The UGT1A gene family encodes pivotal enzymes that play an important role in pharmacology and toxicology, contributing to interindividual differences in drug disposition and cancer risk [8]. Accumulating evidence has demonstrated the differential expression of the UGT1A gene family in a total of 28 tissues, including the pancreas [29–32]. The most important function of the UGT1A gene family is glucuronidation, which provides protection from environmental toxins and contributes to the clearance of a large proportion of commonly used drugs [8]. In this study, we used several large public databases to perform a comprehensive analysis of the relationships between the UGT1A gene family members and the pathogenesis and progression of PC.

First, we explored the mRNA expression of the UGT1A gene family members and their correlation with the pathological stage in PC. We found that the transcriptional levels of UGT1A1/3/6/8/9/10 in PC tissues were significantly higher than those in normal tissues. These results were then validated using five pairs of PC tumor tissues and adjacent nontumor tissues. A significant correlation was found between the expression of UGT1A1/6/10 and the pathological stage of PC. The expression of UGT1A1, UGT1A6, and UGT1A10 increased as the tumor progressed, suggesting that the UGT1A gene family plays an important role in the tumorigenesis and progression of PC.

Second, we explored the function of differentially expressed UGT1A gene family members using GO enrichment analysis and KEGG pathway enrichment analysis. The results showed that the UGT1A gene family members were mainly enriched in glucuronidation and metabolic regulation functions, and the functions of these genes were primarily related to ascorbate and aldarate metabolism, pentose and glucuronate interconversions, porphyrin and chlorophyll metabolism, drug metabolism-other enzymes, steroid hormone biosynthesis, drug metabolism-cytochrome P450, metabolism of xenobiotics by cytochrome P450, chemical carcinogenesis, and metabolic pathways. Glucuronidation of drugs via elevation of UGT1As correlates with clinical resistance [33]. Glucuronidation plays an important role in clearing metabolites and drug detoxification [34, 35]. A study showed that “turning on” UGT1A gene family activity could be the basis of a multidrug resistance mechanism [36]. These data suggest that the UGT1A gene family members are potential drug therapeutic targets.

Third, to evaluate the value of differentially expressed UGT1A gene family members in the progression of PC, we assessed the correlation between the differentially expressed UGT1A gene family and clinical outcomes. The results showed that patients with lower transcriptional levels of UGT1A1/4/5/6/10 were associated with a significantly better prognosis, whereas patients with high transcriptional levels of UGT1A1, UGT1A4, and UGT1A10 were significantly associated with shorter RFS. A study showed that the UGT1A gene family plays an important role in the treatment of modified FOLFIRINOX, improving the long-term survival of patients with PC [12]. These data suggest that the UGT1A gene family is a potential prognostic target in patients with PC.

Because multiple UGT1A gene family members were significantly differentially expressed in PC, we explored the genetic alteration and carcinogenic mechanism of the UGT1A gene family members. The genetic alterations in UGT1A gene family members in PC patients varied from 0.4 to 1.1% for individual genes. Studies have shown that the genetic alteration of UGT1A gene family members reduces the ability of UGT to use UDP-GlcUA and CYP3A4-mediated enhancement of catalytic activity [37]. Furthermore, evaluation of the OS, DFS, and PFS among the altered and unaltered groups did not reveal any significant differences.

We also sought to characterize the TF targets of the differentially expressed UGT1A gene family members and found that HNF1A, AHR, and CDX2 are the three key TFs associated with the regulation of the UGT1A gene family.

Recently, an increasing number of studies have shown that the tumor immune microenvironment is of great importance in predicting clinical outcomes and developing immunotherapy in pancreatic cancer [38]. Xu et al. combined the analysis of macrophages and immune checkpoints as an enhanced indicator of survival in patients with PC, implicating the value of the combination therapy [39]. Our results showed that the expression of the UGT1A gene family was negatively correlated with the number of infiltrating macrophages. B cells are special features of pancreatic tumors [40], and studies have shown that a high B cell infiltrate is associated with a better prognosis in patients with PC [41, 42]. Importantly, our results also showed that the expression levels of UGT1A1/3/8/9/10 were positively correlated with the activities of tumor-infiltrating immune cells, especially B cells. These results indicate that the UGT1A gene family may noticeably correlate with immune cell infiltration, which may help us better understand the immune microenvironment of patients with PC.

Our study has some limitations. Analysis at the transcriptional level can reflect some aspects of immune status, but not global changes. Moreover, another independent cohort and in vitro or in vivo studies should be performed to validate our results.

## 5. Conclusions

Overall, our results suggest that the UGT1A gene family may serve as a potential target and prognostic biomarker for patients with PC. However, future studies are required to validate our findings and thus promote the clinical utility of the UGT1A gene family in PC.

## Figures and Tables

**Table 1:** The mRNA expression levels of the UGT1A family in PC and normal tissues (Oncomine).

Genes	Cancer type	Fold change	*P* value	*t*-test	References
UGT1A1	Pancreatic carcinoma	12.962	4.66*E* − 7	6.211	Pei 2009

UGT1A3	Pancreatic carcinoma	7.861	5.17*E* − 6	5.388	Pei 2009

UGT1A6	Pancreatic adenocarcinoma	12.025	2.76*E* − 5	7.063	Logsdon2003
	Pancreatic carcinoma	6.999	8.41*E* − 6	5.183	Pei 2009

UGT1A8	Pancreatic carcinoma	2.626	3.51*E* − 5	4.405	Pei 2009

UGT1A9	Pancreatic carcinoma	6.065	1.52*E* − 5	4.956	Pei 2009

**Table 2:** Key regulated factor of the UGT1A family in PC (TRRUST).

TFs	Targets	Mode of regulation	References (PMID)
HNF1A	UGT1A1/UGT1A7/UGT1A8/UGT1A9/UGT1A10	Activation/unknown/activation/activation/activation	18172616/20406851/15044625/15044625/15044625
AHR	UGT1A1/UGT1A6	Activation/unknown	18172616/9466822
CDX2	UGT1A8/UGT1A10	Activation/activation	15044625/15044625

## Data Availability

Data in this study can be acquired from the corresponding author on reasonable request and from supplementary files.

## Supplementary Materials

Supplementary Materials Supplementary 1: the mRNA expression levels of UGT1A gene family members in pancreatic cancer (Oncomine). Supplementary 2: the relative level of UGT1A gene family members in pancreatic cancer. Supplementary 3: coexpression, physical interactions, and predicted and shared protein domain similarity of UGT1A gene family members and top 20 genes closely related to the UGT1A gene family in patients with PC (GeneMANIA and R software). Supplementary 4: pathway interactions of UGT1A gene family members and top 20 genes closely related to the UGT1A gene family in patients with PC (GeneMANIA and R software). Supplementary 5: the primer sequences of UGT1A gene family members. Supplementary 6: functional enrichment analysis (top 10) of UGT1A gene family members in patients with pancreatic cancer (DAVID 6.8).
