# Supplementary material for: UGT1A Gene Family Members Serve as Potential Targets and Prognostic Biomarkers for Pancreatic Cancer
Source: Biomed Res Int. 2021 Sep 20;2021:6673125. doi: 10.1155/2021/6673125 (PMC8478536; doi:10.1155/2021/6673125)
Supplement: Supplementary Materials — Supplementary 1: the mRNA expression levels of UGT1A gene family members in pancreatic cancer (Oncomine). Supplementary 2: the relative level of UGT1A gene family members in pancreatic cancer. Supplementary 3: coexpression, physical interactions, and predicted and shared protein domain similarity of UGT1A gene family members and top 20 genes closely related to the UGT1A gene family in patients with PC (GeneMANIA and R software). Supplementary 4: pathway interactions of UGT1A gene family members and top 20 genes closely related to the UGT1A gene family in patients with PC (GeneMANIA and R software). Supplementary 5: the primer sequences of UGT1A gene family members. Supplementary 6: functional enrichment analysis (top 10) of UGT1A gene family members in patients with pancreatic cancer (DAVID 6.8). [file 6673125.f1.docx]

**Supplementary Files**


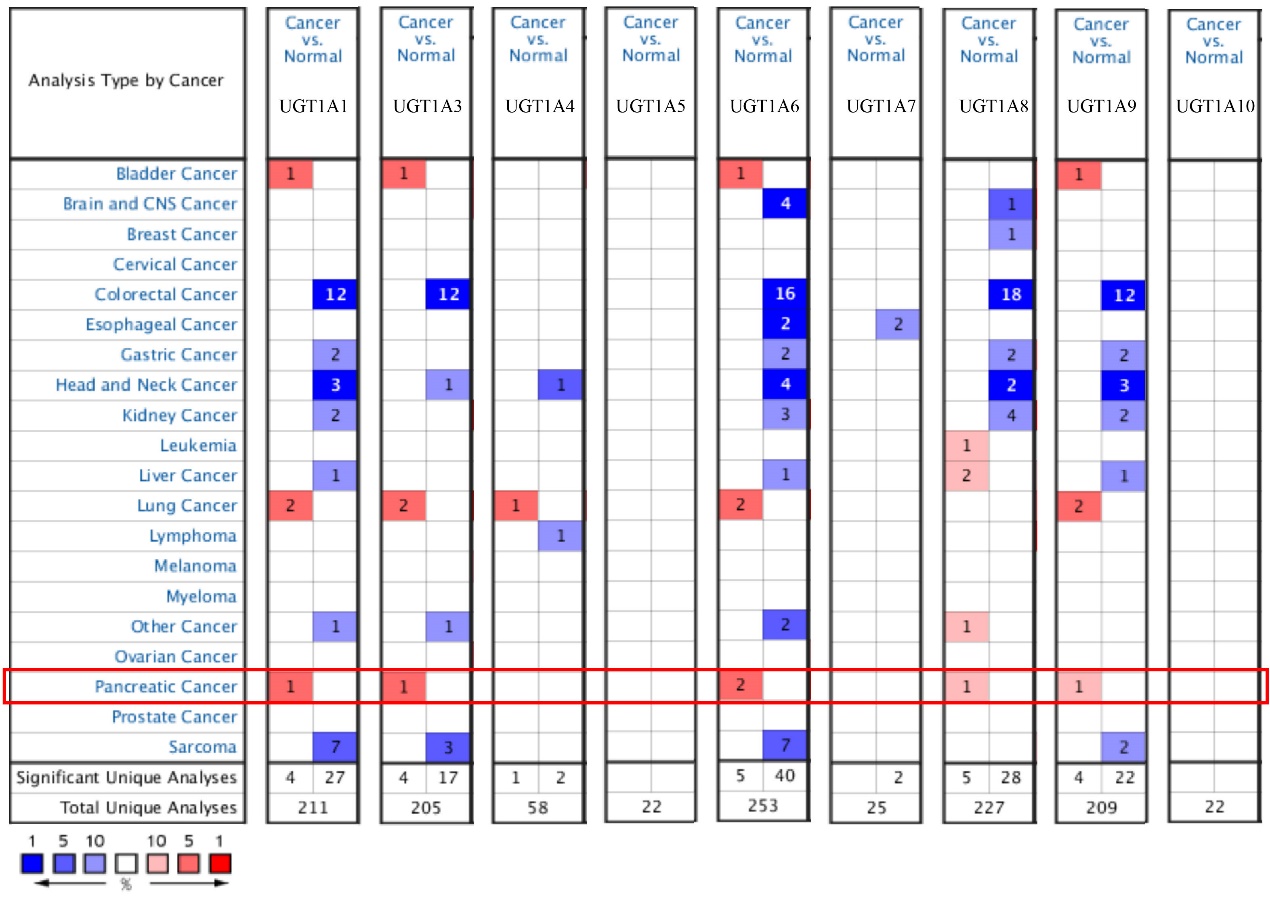


**Supplementary 1:** The mRNA expression levels of UGT1A gene family members in pancreatic cancer (Oncomine). The figure shows the numbers of datasets which has statistically significant mRNA over-expression(red) or downregulated expression (blue) of UGT1A gene family members.


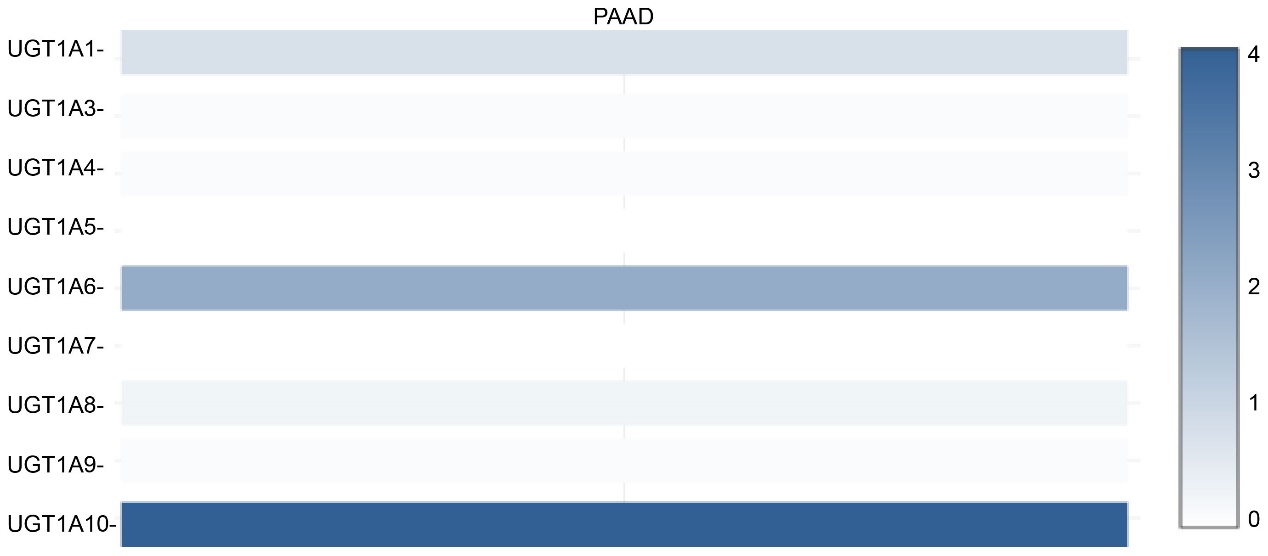


**Supplementary 2:** The relative level of UGT1A gene family members in pancreatic cancer.


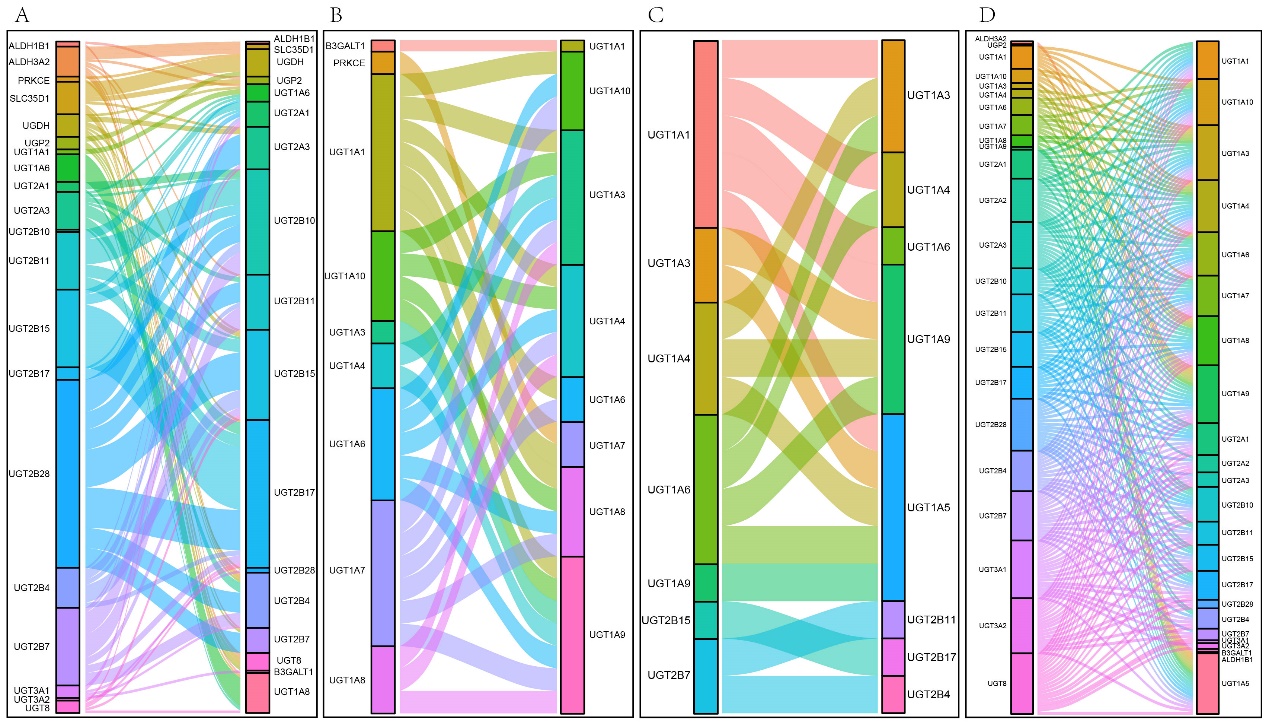


**Supplementary 3:** Co-expression, physical interactions, predicted and share protein domain similarity of UGT1A gene family members and top 20 genes closely related UGT1A gene family in patients with PC (GeneMANIA and R software). (A): co-expression; (B): physical interactions; (C): predicted; (D): share protein domain.

**
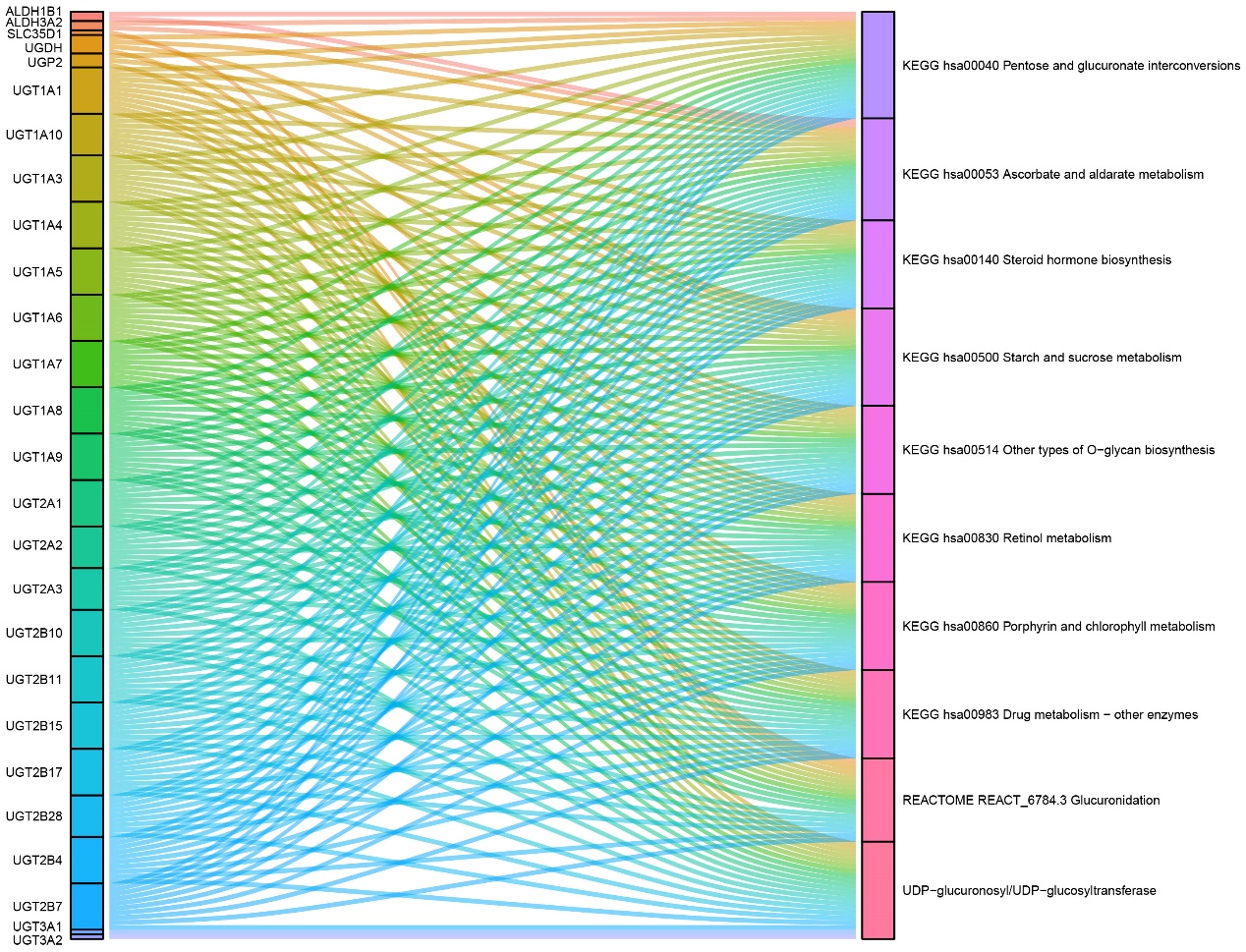
**

**Supplementary 4**: Pathway interactions of UGT1A gene family members and top 20 genes closely related UGT1A gene family in patients with PC (GeneMANIA and R software).

**Supplementary 5**: The primer sequences of UGT1A gene family members.

| **Gene name** | **Primer sequences (from 5’ to 3’)** |
| --- | --- |
| UGT1A1-F | ACTGCCTTCACCAAAATCCACTA |
| UGT1A1-R | TACCGCCACAGGACTGTCTGA |
| UGT1A3-F | AGAGGTGTCAGTGGTGGATATTCT |
| UGT1A3-R | TAATGTAGGCTTCAAATTCCTGAGA |
| UGT1A4-F | GAGGTGTCAGTGGTGGATCT |
| UGT1A4-R | TGTAGGCTTCAAATTCCTGAGA |
| UGT1A5-F | AGAGAGGTGTCAGTGGTGGAT |
| UGT1A5-R | TGTAGGCTTCAAATTCCTGAGAT |
| UGT1A6-F | AGAAGGTCTCTGTTTGGCTGT |
| UGT1A6-R | TGTAGGCTTCAAATTCCTGAGACA |
| UGT1A7-F | CCTGTCACGGCATATGATCT |
| UGT1A7-R | AGGCTTCAAATTCCATAGGCA |
| UGT1A8-F | GGTCTTCGCCAGGGGAATAGC |
| UGT1A8-R | GTTCGCAAGATTCGATGGTCG |
| UGT1A9-F | GGAGGAACATTTATTATGCCACCG |
| UGT1A9-R | GTTCGCAAGATTCGATGGTCG |
| UGT1A10-F | CCTCTTTCCTATGTCCCCAATGA |
| UGT1A10-R | CCTTAGTCTCCATGCGCTTTGC |
| GAPDH-F | CACCCACTCCTCCACCTTTG |
| GAPDH-R | CCACCACCCTGTTGCTGTAG |

**Supplementary 6:** Functional enrichment analysis (top 10) of UGT1A gene family members in patients with pancreatic cancer (DIVID 6.8)

| **Category** | **Terms** | **Genes** | **P-Value** | **FDR** |
| --- | --- | --- | --- | --- |
| GOTERM_BP | GO:0052697~xenobiotic glucuronidation | UGT1A7, UGT1A10, UGT1A6, UGT1A9, UGT1A8, UGT1A3, UGT1A5, UGT1A4, UGT1A1 | 5.75E-29 | 5.06E-26 |
| GOTERM_BP | GO:0052696~flavonoid glucuronidation | UGT1A7, UGT1A10, UGT1A6, UGT1A9, UGT1A8, UGT1A3, UGT1A5, UGT1A4, UGT1A1 | 2.04298E-24 | 1.8E-21 |
| GOTERM_BP | GO:0008152~metabolic process | UGT1A7, UGT1A10, UGT1A6, UGT1A9, UGT1A8, UGT1A3, UGT1A5, UGT1A4, UGT1A1 | 8.48975E-17 | 9.99E-14 |
| GOTERM_BP | GO:1904224~negative regulation of glucuronosyltransferase activity | UGT1A7, UGT1A10, UGT1A6, UGT1A9, UGT1A8, UGT1A3, UGT1A4, UGT1A1 | 8.57849E-25 | 7.54E-22 |
| GOTERM_BP | GO:2001030~negative regulation of cellular glucuronidation | UGT1A7, UGT1A10, UGT1A6, UGT1A9, UGT1A8, UGT1A3, UGT1A4, UGT1A1 | 8.57849E-25 | 7.54E-22 |
| GOTERM_BP | GO:0045922~negative regulation of fatty acid metabolic process | UGT1A7, UGT1A10, UGT1A6, UGT1A9, UGT1A8, UGT1A3, UGT1A4, UGT1A1 | 3.86009E-24 | 3.39E-21 |
| GOTERM_BP | GO:0052695~cellular glucuronidation | UGT1A7, UGT1A10, UGT1A6, UGT1A9, UGT1A8, UGT1A3, UGT1A4, UGT1A1 | 1.22614E-21 | 1.08E-18 |
| GOTERM_BP | GO:0009813~flavonoid biosynthetic process | UGT1A7, UGT1A10, UGT1A6, UGT1A3, UGT1A5, UGT1A4, UGT1A1 | 3.48276E-17 | 3.06E-14 |
| GOTERM_BP | GO:0051552~flavone metabolic process | UGT1A7, UGT1A10, UGT1A9, UGT1A8, UGT1A1 | 3.16912E-13 | 2.79E-10 |
| GOTERM_BP | GO:0042573~retinoic acid metabolic process | UGT1A7, UGT1A9, UGT1A8, UGT1A3, UGT1A1 | 2.11184E-11 | 1.86E-08 |
|  |  |  |  |  |
| GOTERM_CC | GO:0016021~integral component of membrane | UGT1A7, UGT1A10, UGT1A6, UGT1A9, UGT1A8, UGT1A3, UGT1A5, UGT1A4, UGT1A1 | 4.13407E-05 | 0.02263 |
| GOTERM_CC | GO:0005783~endoplasmic reticulum | UGT1A7, UGT1A10, UGT1A6, UGT1A9, UGT1A8, UGT1A3, UGT1A4, UGT1A1 | 2.99765E-09 | 1.64E-06 |
| GOTERM_CC | GO:0005789~endoplasmic reticulum membrane | UGT1A7, UGT1A10, UGT1A6, UGT1A9, UGT1A8, UGT1A3, UGT1A4, UGT1A1 | 3.97024E-09 | 2.17E-06 |
|  |  |  |  |  |
| GOTERM_MF | GO:0015020~glucuronosyltransferase activity | UGT1A7, UGT1A10, UGT1A6, UGT1A9, UGT1A8, UGT1A3, UGT1A5, UGT1A4, UGT1A1 | 2.62863E-23 | 1.69E-20 |
| GOTERM_MF | GO:0001972~retinoic acid binding | UGT1A7, UGT1A10, UGT1A6, UGT1A9, UGT1A8, UGT1A3, UGT1A4, UGT1A1 | 2.53108E-20 | 1.63E-17 |
| GOTERM_MF | GO:0019899~enzyme binding | UGT1A7, UGT1A10, UGT1A6, UGT1A9, UGT1A8, UGT1A3, UGT1A4, UGT1A1 | 8.58974E-12 | 5.53E-09 |
| GOTERM_MF | GO:0046982~protein heterodimerization activity | UGT1A7, UGT1A10, UGT1A6, UGT1A9, UGT1A8, UGT1A3, UGT1A4, UGT1A1 | 8.99228E-11 | 5.79E-08 |
| GOTERM_MF | GO:0042803~protein homodimerization activity | UGT1A7, UGT1A10, UGT1A6, UGT1A9, UGT1A8, UGT1A3, UGT1A4, UGT1A1 | 2.11825E-09 | 1.36E-06 |
| GOTERM_MF | GO:0016758~transferase activity, transferring hexosyl groups | UGT1A7, UGT1A6, UGT1A9, UGT1A8, UGT1A3, UGT1A4, UGT1A1 | 5.16266E-16 | 3.55E-13 |
| GOTERM_MF | GO:0004857~enzyme inhibitor activity | UGT1A7, UGT1A9, UGT1A8, UGT1A1 | 2.8193E-07 | 0.000181 |
| GOTERM_MF | GO:0005496~steroid binding | UGT1A8, UGT1A1 | 0.012726674 | 7.910086 |
| GOTERM_MF | GO:0005080~protein kinase C binding | UGT1A7, UGT1A10 | 0.02159731 | 13.10524 |
| GOTERM_MF | GO:0008144~drug binding | UGT1A7, UGT1A8 | 0.035461613 | 20.72853 |
|  |  |  |  |  |
| KEGG_PATHWAY | hsa00053: Ascorbate and aldarate metabolism | UGT1A7, UGT1A10, UGT1A6, UGT1A9, UGT1A8, UGT1A3, UGT1A5, UGT1A4, UGT1A1 | 1.79248E-20 | 1.08E-17 |
| KEGG_PATHWAY | hsa00040: Pentose and glucuronate interconversions | UGT1A7, UGT1A10, UGT1A6, UGT1A9, UGT1A8, UGT1A3, UGT1A5, UGT1A4, UGT1A1 | 1.121E-19 | 6.73E-17 |
| KEGG_PATHWAY | hsa00860: Porphyrin and chlorophyll metabolism | UGT1A7, UGT1A10, UGT1A6, UGT1A9, UGT1A8, UGT1A3, UGT1A5, UGT1A4, UGT1A1 | 9.52971E-19 | 5.72E-16 |
| KEGG_PATHWAY | hsa00983: Drug metabolism-other enzymes | UGT1A7, UGT1A10, UGT1A6, UGT1A9, UGT1A8, UGT1A3, UGT1A5, UGT1A4, UGT1A1 | 2.10676E-18 | 1.27E-15 |
| KEGG_PATHWAY | hsa00140: Steroid hormone biosynthesis | UGT1A7, UGT1A10, UGT1A6, UGT1A9, UGT1A8, UGT1A3, UGT1A5, UGT1A4, UGT1A1 | 1.54761E-17 | 9.29E-15 |
| KEGG_PATHWAY | hsa00830: Retinol metabolism | UGT1A7, UGT1A10, UGT1A6, UGT1A9, UGT1A8, UGT1A3, UGT1A5, UGT1A4, UGT1A1 | 3.57367E-17 | 2.15E-14 |
| KEGG_PATHWAY | hsa00982:D rug metabolism-cytochrome P450 | UGT1A7, UGT1A10, UGT1A6, UGT1A9, UGT1A8, UGT1A3, UGT1A5, UGT1A4, UGT1A1 | 5.96828E-17 | 6.66E-14 |
| KEGG_PATHWAY | hsa00980: Metabolism of xenobiotics by cytochrome P450 | UGT1A7, UGT1A10, UGT1A6, UGT1A9, UGT1A8, UGT1A3, UGT1A5, UGT1A4, UGT1A1 | 1.21686E-16 | 6.66E-14 |
| KEGG_PATHWAY | hsa05204: Chemical carcinogenesis | UGT1A7, UGT1A10, UGT1A6, UGT1A9, UGT1A8, UGT1A3, UGT1A5, UGT1A4, UGT1A1 | 2.34044E-16 | 1.33E-13 |
| KEGG_PATHWAY | hsa01100: Metabolic pathways | UGT1A7, UGT1A10, UGT1A6, UGT1A9, UGT1A8, UGT1A3, UGT1A5, UGT1A4, UGT1A1 | 9.54112E-07 | 0.000573 |
